# Modifying Reduced Graphene Oxide by Conducting Polymer Through a Hydrothermal Polymerization Method and its Application as Energy Storage Electrodes

**DOI:** 10.1186/s11671-019-3051-6

**Published:** 2019-07-09

**Authors:** Shiyuan Li, Yan Chen, Xin He, Xiling Mao, Yujiu Zhou, Jianhua Xu, Yajie Yang

**Affiliations:** 1 State Key Laboratory of Electronic Thin Films and Integrated Devices, School of Optoelectronic Science and Engineering, University of Electronic Science and Technology of China (UESTC), Chengdu, 610054 People’s Republic of China; 2 College of Optoelectronic Technology, University of Information Technology, Chengdu, 610225 People’s Republic of China

**Keywords:** Hydrothermal polymerization, Reduced graphene oxide, Conducting polymer, Supercapacitor

## Abstract

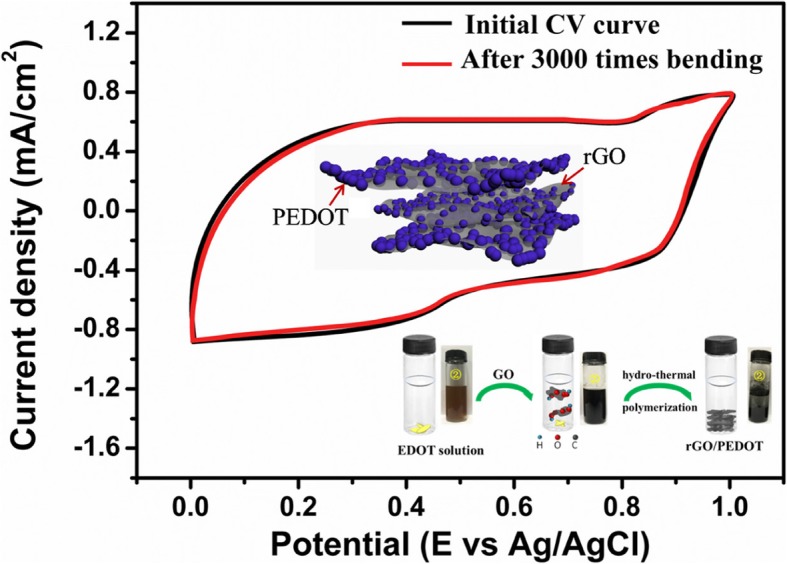

## Research Highlights

In this paper, as an easy and facile way, the functional groups on graphene oxide (GO) were directly employed as an oxidizer to trigger the polymerization of 3,4-ethylenedioxythiophene (EDOT) and the GO nanosheets were reduced into rGO accordingly in an aqueous environment. The high specific capacitance and high conductivity of composite electrode were achieved through this simple method. The composite electrode also showed good cycling stability and flexibility, which exhibited a promising future to construct high-performance flexible devices.

## Introduction

In recent years, conducting polymers (CPs) that are highly conductive and electrochemically active have been focused on some promising applications such as solar cell [1–3], sensors [4–8], energy storage devices [9–11], and bioelectronics [12–14]. The energy storage device, such as supercapacitors, has employed the conducting polymer as high-efficiency electrodes, which can afford high pseudocapacitance due to its reversible oxidation/reduction process in electrolytes [15–18]. In order to obtain conducting polymer-based electrochemical electrode with high stability, incorporating conducting polymer with carbon materials and metal oxide materials with nanostructure has been extensively investigated [19–22]. Due to the highly stable performance of these materials, the significant improvement of electrochemical stability of conducting polymer has been achieved, which also results in constructing devices with excellent energy storage performance [23, 24]. In these aspects, incorporating conducting polymer with carbon nanomaterials is more suitable to obtain composite electrodes with high energy density and power density simultaneously. Moreover, compared with inorganic materials, the excellent flexibility of conducting polymer also benefits for constructing flexible electrode, even the flexible devices for wearable electronic systems [25–29].

The conducting polymer/carbon nanomaterial electrodes exhibit high energy density due to the combination of pseudocapacitance and electric double layer capacitance (EDLC) [15, 30–32]. Hence, the optimized synergistic effect between two components is vital to obtain a composite electrode with high specific capacitance and high stability, which means that the electrode performance depends greatly on the preparation method. In order to obtain heterostructured structures, different methods, such as physical mixing [33–35], electrochemical polymerization [36], and chemical in situ polymerization with oxidant [37–40], were employed to prepare the conducting polymer and its composite as supercapacitor electrodes. As for the physical mixing method, well distribution and alloying of two components need to be considered carefully to avoid the phase separation during the long time cycling. As well as chemical in situ polymerization with oxidant in a solution or gaseous environment, due to the polymerization of monomer on carbon nanomaterials with a thermodynamic process, excellent synergistic effect has been confirmed in these composite materials [41, 42]. However, both these in situ polymerization methods are suffered with difficulty to wash out the excess oxidant, which will take great influence on the morphology and related performance of as-prepared composites [43, 44]. Therefore, it has great demands to prepare conducting polymer/carbon nanomaterials with an oxidant-free method, which will produce composite through a simple and facile way, coupling the advantages of carbon nanomaterials and conducting polymer.

In this paper, based on solution processability of GO and functional groups on GO sheets, conducting polymer poly (3,4-ethylenedioxythiophene) (PEDOT) was anchoring on GO sheets through a simple hydrothermal polymerization method. The functional groups on GO sheets play a role as an oxidant to trigger the polymerization of 3,4-ethylenedioxythiophene (EDOT) monomer, and a reduced graphene oxide (rGO)/PEDOT nanocomposite was obtained. Due to this oxidant-free method, the ultrathin and ultramolecular modification of conducting polymer on rGO nanosheets was achieved. The resultant rGO/PEDOT nanocomposite has been studied as promising electrochemical electrode materials for supercapacitor applications.

## Materials and Methods

### Materials

Graphite flakes used for GO preparation were purchased from Sigma-Aldrich. GO was synthesized from natural graphite flakes prepared through Hummer’s method [45]. For chemical in situ polymerization, EDOT monomer was purchased from Bayer Company. Other chemical reagents with analytical grade were purchased from Chengdu Kelong Chemical Reagent Company and used as received.

### Preparation of rGO/PEDOT Nanocomposite

**Fig. 1:**
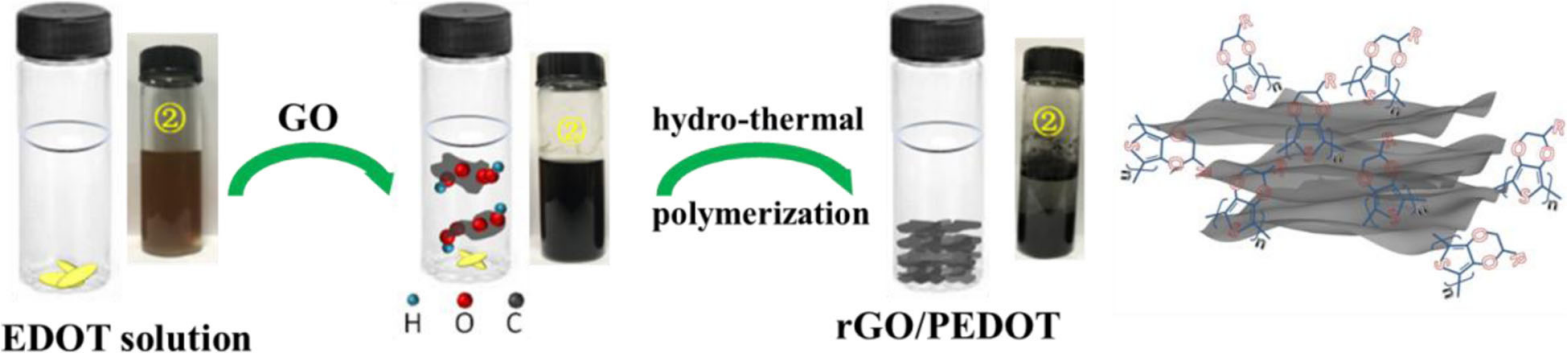
The schematic diagram of hydrothermal polymerization of rGO/PEDOT

Fifty microliters of EDOT monomer was introduced into 50 ml DI water with magnetic stirring for 2 h to prepare EDOT solution. The stable GO dispersion was prepared by introducing 1.5 g of GO sheets into 30 ml DI water and subjected to magnetic stirring for 1 h followed by centrifugation at 2500 rpm. Subsequently, 10 ml EDOT solution was dropwise into the GO solution with magnetic stirring at 60 °C for 6 h, and then, the reaction temperature was increased to 90 °C at least 2 h for the further polymerization of EDOT and reduction of GO (rGO/PEDOT1). The schematic diagram of EDOT polymerization and reduction of GO is presented in Fig. 1. In order to compare the influence of GO contents on the performance of obtained composite, different masses of GO were added into DI water for the hydrothermal reaction. So, as mentioned in the preparation process above, all the experimental parameters are the same except the GO mass added into DI water. Accordingly, a 3 g, a 4 g, and a 4.5 g GO sheets produced the rGO/PEDOT2, rGO/PEDOT3, and rGO/PEDOT4 composites, respectively. The pure rGO was obtained through the hydrothermal method mentioned above.

### Characterization and Electrochemical Performance Test

Surface morphology of GO and rGO/PEDOT was investigated using a scanning electron microscope (SEM, Hitachi, Model S-2400). UV-Vis spectrum of the film was characterized by a UV-1700 spectrometer (SHIMADZU). FT-IR spectrum was obtained with an ALPHA analysis instrument (Germany). For the conductivity test, the obtained rGO and composite powder were compressed into a cylinder shape with the size of 5 mm (radius) × 2 mm (height) and tested in room temperature. Electrical conductivity was tested by a SX193 Four-Probe testing system (Baishen, Suzhou, China). XPS spectra were carried out by using an Escalab 250Xi photoelectron spectrometer (Thermo Fisher Scientific, USA). Raman spectra were recorded on an Alpha300 model with a 532-nm laser (WITec, Germany). Electrochemical performance was investigated by using a CHI600 electrochemistry workstation (Chenhua, Shanghai, China). Cyclic voltammetry (CV), galvanostatic charge–discharge (GCD), and electrochemical impedance spectroscopy (EIS) were performed with 1 mol/L H_2_SO_4_ aqueous electrolyte using a platinum sheet as the counter electrode and Ag/AgCl as the reference electrode. All the measurements were performed at ambient temperature.

## Results and Discussion

**Fig. 2:**
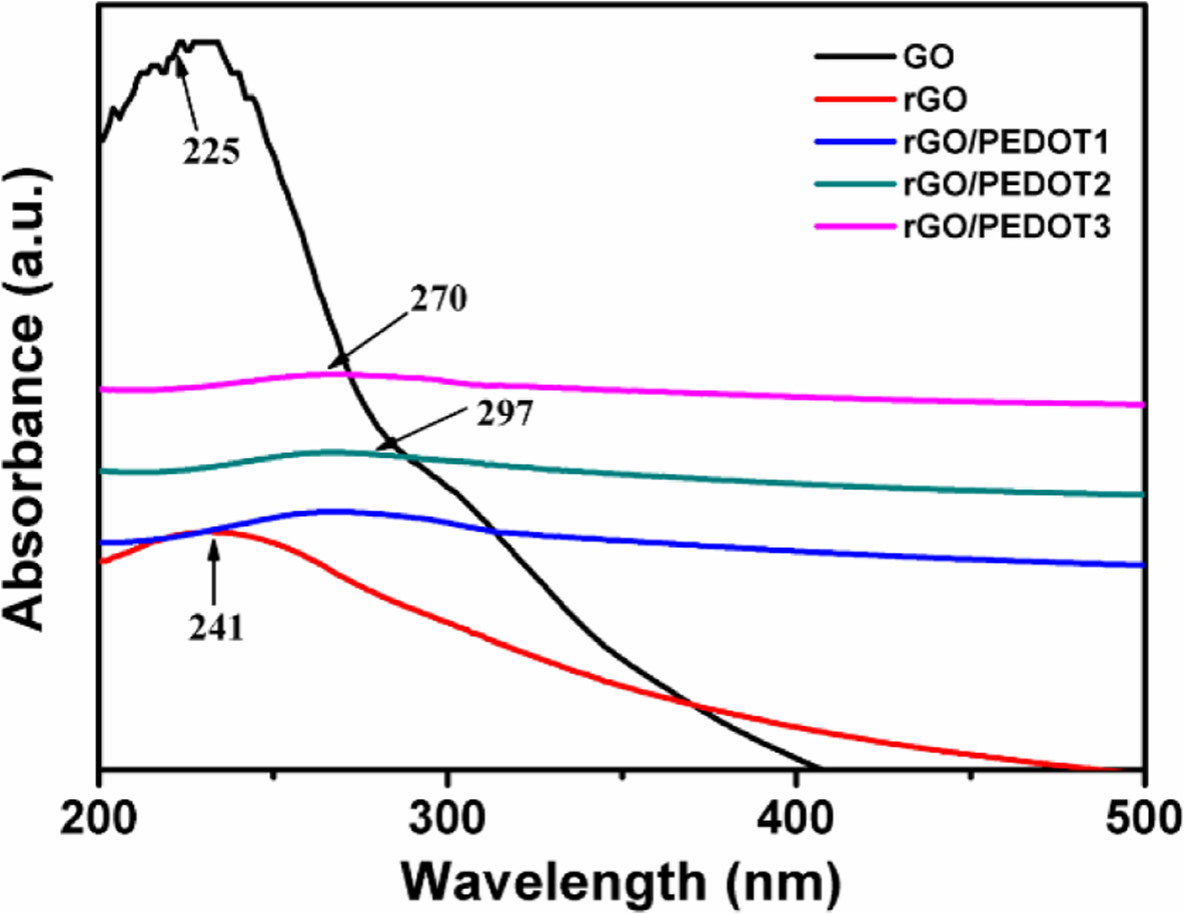
UV-Vis spectrum of GO, hydrothermal-treated rGO, and hydrothermal-polymerized rGO/PEDOT

The UV-Vis spectrum of GO, hydrothermal-treated rGO, and hydrothermal-polymerized rGO/PEDOT is shown in Fig. 2. The pure GO shows two main absorption bands, and the maximum peak presents in 225 nm, attributing to the *π*→π* transition of C=C bond. A weak absorption peak at 297 nm resulting from the *n*→π* transition of C=O bond is also observed [46]. After hydrothermal treatment, the obtained rGO shows different characteristic peaks with the evidence that the 225-nm peak shifts to 241 nm and the 297-nm peak disappears. This result indicates that the hydrothermal treatment partly removes the functional groups on the GO sheets. The color of GO solution also changes from the yellow to deep black after this hydrothermal treatment (as shown in Fig. 1). From Fig. 2, we can see that rGO/PEDOT composite shows an absorption peak at 270 nm, which comes from the *π*→π* transition of rGO [46]. Furthermore, a broad absorption peak from 450 nm to near-infrared wavelength appears in the spectrum due to the typical absorption of polarons and bipolarons in polymerized PEDOT [24]. After the addition of monomer EDOT into GO solution, hydrothermal treatment triggers the polymerization of EDOT and the GO is reduced to rGO accordingly, and this oxidant-free polymerization method produces the rGO/PEDOT composite in an aqueous environment successfully. It also can be seen from Fig. 2 that with the increase of GO mass during the hydrothermal polymerization, the obtained rGO/PEDOT shows more obvious shift of absorption peak, rising from *π*→π* transition to longer wavelength than pure rGO. This result reveals that more functional groups in GO were employed to trigger the polymerization of EDOT in the oxidant-free condition.

**Fig. 3:**
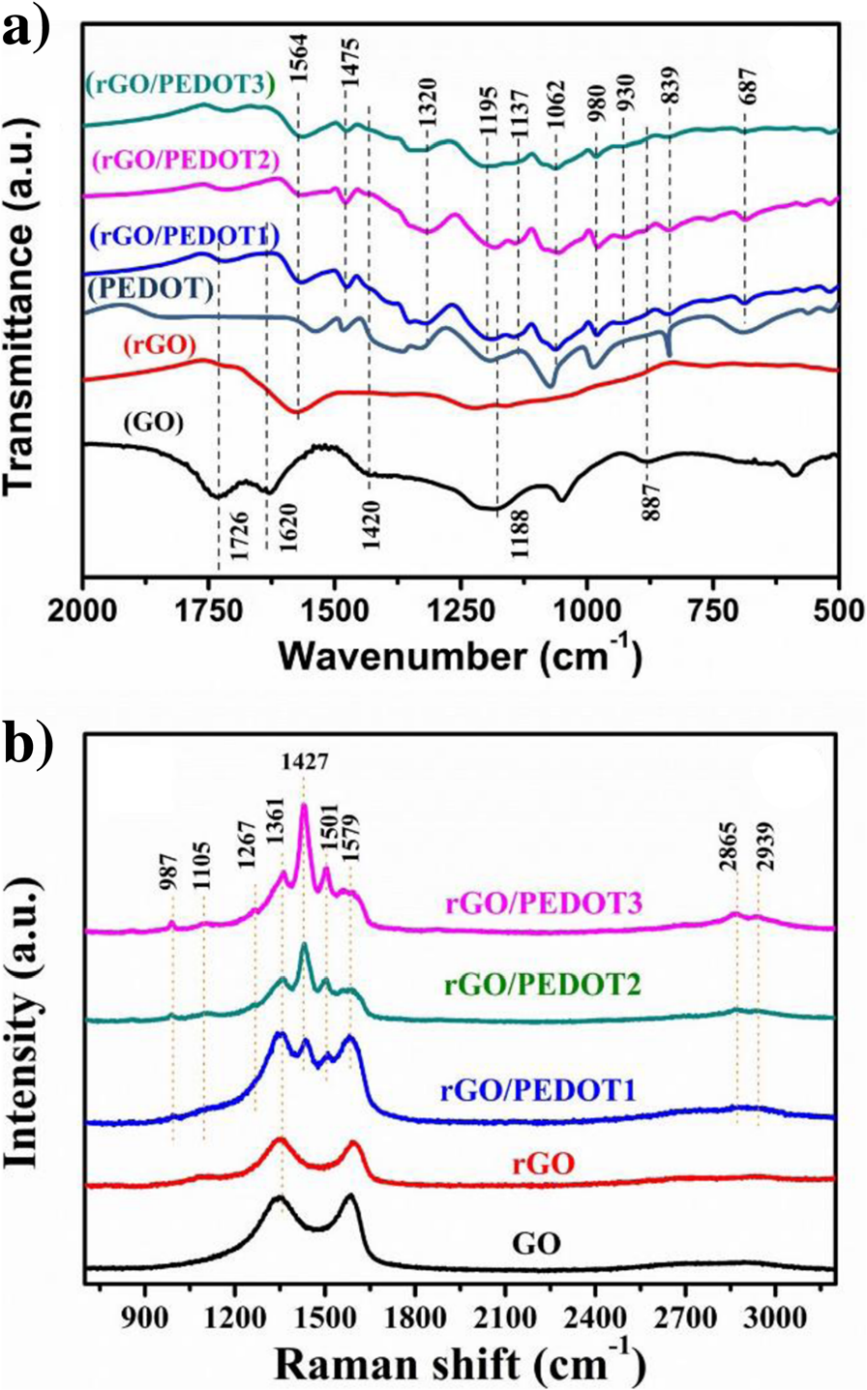
**a** FT-IR spectra of GO, hydrothermal-treated rGO, and hydrothermal-polymerized rGO/PEDOT. **b** Raman spectra of GO, hydrothermal-treated rGO, and hydrothermal-polymerized rGO/PEDOT

The FT-IR and Raman analysis were employed to further confirm what functional groups trigger the polymerization of EDOT monomer during the hydrothermal treatment. Figure 3a shows the FT-IR spectrum of GO, hydrothermal-treated rGO, and different rGO/PEDOT composites. The absorption peaks at 3395 cm^−1^ and 1726 cm^−1^ come from the stretching vibration of C–OH bond and C=O bond located at GO edge. The 1620 cm^−1^ peak represents the vibration absorption of C=C bond and 1420 cm^−1^ peak rising from the deformed –OH bond on the GO plane [47]. The 1188 cm^−1^ peak results from the vibration of C–O bonds [48]. After the hydrothermal treatment, the peaks located at 3395 cm^−1^ and 1419 cm^−1^ were weakened obviously, indicating the partly removal of the –OH group. The peak density located at 1726 cm^−1^ and 887 cm^−1^ resulted from –OH and C–O–C absorption, which is also weaken distinctly and even disappears, indicating that the above functional groups were reduced and are realized as possible active sites for the polymerization of EDOT [49, 50]. The reduction of GO is also confirmed with the evidence that the peak density of 1571 cm^−1^ enhances obviously, resulting from the C=C bond absorption, which indicates the recovery of plain conjugated structure of rGO [51].

After the hydrothermal treatment of EDOT/GO solution, the FT-IR spectrum of composite shows characteristic peaks of PEDOT polymer. It can be seen that the spectrum of composite shows series peaks at 687 cm^−1^, 839 cm^−1^, 930 cm^−1^, and 980 cm^−1^, which rise from the vibration of C-S bond in PEDOT [52, 53]. In addition, the peaks located at 1026 cm^−1^, 1137 cm^−1^, and 1195 cm^− 1^ were also observed due to the stretching absorption of C–O–C bond in alkylenedioxy [54, 55]. Therefore, comparing the FT-IR spectrum of GO with rGO/PEDOT, the distinct disappearance of –OH (located at 1420 cm^−1^) and C–O–C (located at 887 cm^−1^) absorption was observed, indicating that these two functional groups trigger the polymerization of EDOT primarily during hydrothermal treatment [56].

The Raman spectrum of GO, hydrothermal-treated rGO, and hydrothermal-polymerized rGO/PEDOT is shown in Fig. 3b. Compared with pure GO and rGO, the rGO/PEDOT shows new characteristic peaks at 1427 cm^−1^ and 1501 cm^−1^, and these peaks are attributed to the symmetric and asymmetric stretching vibration of C=C bond in PEDOT. The peaks located at 1361 cm^−1^ and 1267 cm^−1^ are attributed to C_β_–C_β_ and C_α_–C_α_ of thiophene ring. The peak located at 987 cm^−1^ rises from the deformed vibration of oxyethylene ring [57]. Two peaks at 2856 cm^−1^ and 2939 cm^−1^ are attributed to doped PEDOT [58–60].

**Fig. 4:**
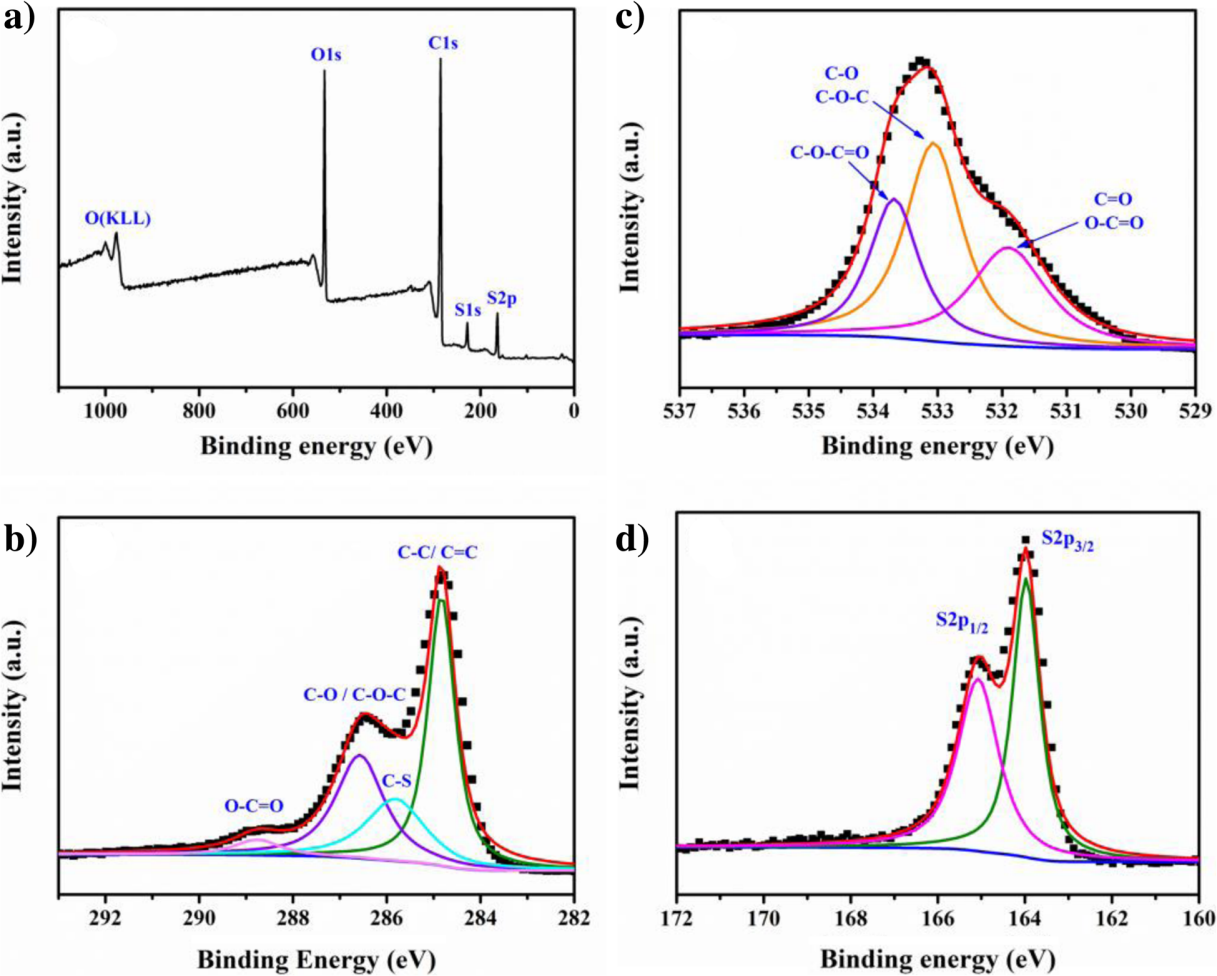
XPS spectra of rGO/PEDOT. **a** Full spectra. **b** C_1s_. **c** O_1s_. **d** S_2p_

In order to further investigate the obtained composite, the XPS analysis is utilized to confirm the reduction of GO and polymerization of EDOT monomer. Figure 4 shows the full XPS spectra of the composite after the hydrothermal treatment. It clearly shows that only C, S, and O elements distribute in the composite. The peak at 980 eV is corresponding to the Auger peak of O element, and the peak at 529~537 eV is attributed to the O_1s_. The peaks at 229 eV and 165 eV result from S_1s_ and S_2p_, respectively. According to the relative contents of C, O, and S in composite, the calculated atom ratio of C/O, C/S, and O/S is 3.42, 14.95, and 4.38, respectively. It has been found that the actual atom ratio of C/S and O/S is higher than pure PEDOT (10.33 and 4.07). We concluded that the -OH and C-O-C trigger the polymerization of EDOT monomer primarily, and some other functional groups in GO will not be reduced in this process. So, the residual groups in rGO will increase the C and S atom ratio accordingly.

**Fig. 5:**
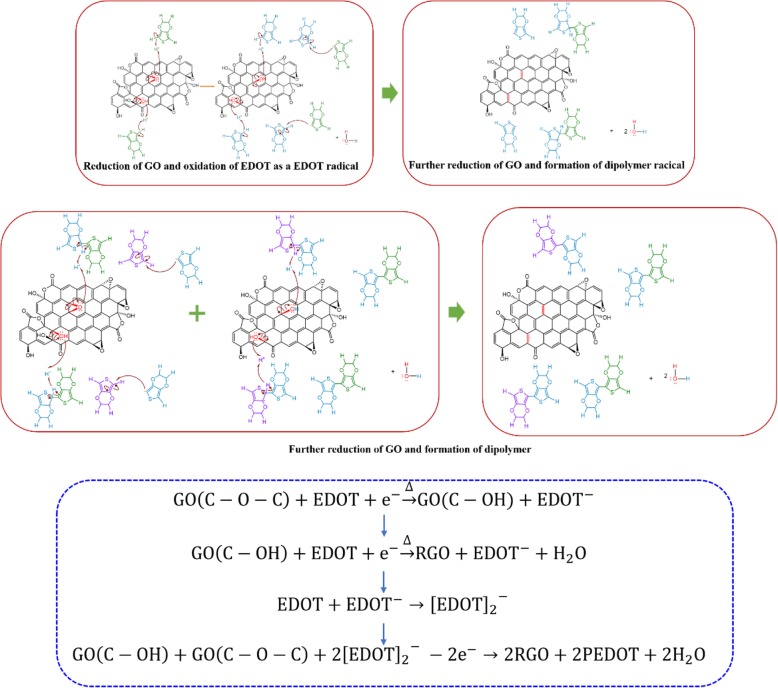
The schematic of hydrothermal polymerization mechanism of rGO/PEDOT

Figure 4 b, c, and d show the fitting C1s, O1s, and S2p peaks of rGO/PEDOT characterized by XPS. In C1s spectra, the high-intensity peak located at 284.8 eV is attributed to the sp2 hybridization in C–C/C=C bonds [61]. The peaks located at 285.8 eV and 288.8 eV rise from the C–S bond in PEDOT and unremoved carboxyl, respectively. The peak present in 286.7 eV results from the C-O-C bond in thiophene ring. As for O1s peaks, the peak located at 533.1 eV is attributed to the epoxy group and C–O–C bond in PEDOT, and peaks at 531.8 eV and 533.7 eV come from the unremoved carboxyl and carbonyl groups at rGO edge. The fitting S2p spectra show two peaks at 163.9 eV and 165.1 eV, which result from the S2p3/2 and S2p1/2 spinning peaks in C–S–C of PEDOT [62]. Moreover, it has been found that compared with the XPS spectrum of pure rGO obtained through hydrothermal method [63], the shift of binding energy about 0.6 eV was observed in composite rGO/PEDOT, and we attribute this to the interaction between the rGO and polymerized PEDOT, which is consistent with the FT-IR results. Combining the FT-IR and XPS analysis, we can further confirm that the oxhydryl and epoxy groups in GO sheets act as primary active sites triggering the polymerization of EDOT monomer, and rGO composite modified by conducting polymer was prepared through simple oxidant-free hydrothermal polymerization method. From the results above, we tentatively explain the polymerization mechanism. For example of C-O-C groups, acting as polymerization trigger at the water bath heating environment, the C-O bond in the GO ring begins to break, making the O atom negatively charged, and the C-H bond in the EDOT begins to break simultaneously. Therefore, under the electrostatic action, the unpaired free electrons in O atom of GO ring oxygen group attract the broken H^+^ from EDOT monomer. Accordingly, an O-H bond at the position of the epoxy group is formed and the EDOT monomer becomes EDOT^−^ radical. By combining with two H^+^ ions, the O atom in the epoxy group breaks away from the GO to form a water molecule, and the new EDOT molecule becomes EDOT^−^ radical again. After this step, the EDOT radical binds to a new neutral EDOT molecule and EDOT dimer radical is formed. At the same time, other EDOT monomers continue to combine with GO and GO is gradually reduced, accompanied by water generation. Then, the EDOT dimer radicals formed by step 2 move to the epoxy group in GO due to electrostatic interaction, then PEDOT with high polymerization degree is formed. The total schematic reaction illustration is shown in Fig. 5. Moreover, a possible connection of the graphene sheets by the EDOT binder through a redox reaction would also improve the conductive performance of composite.

**Fig. 6:**
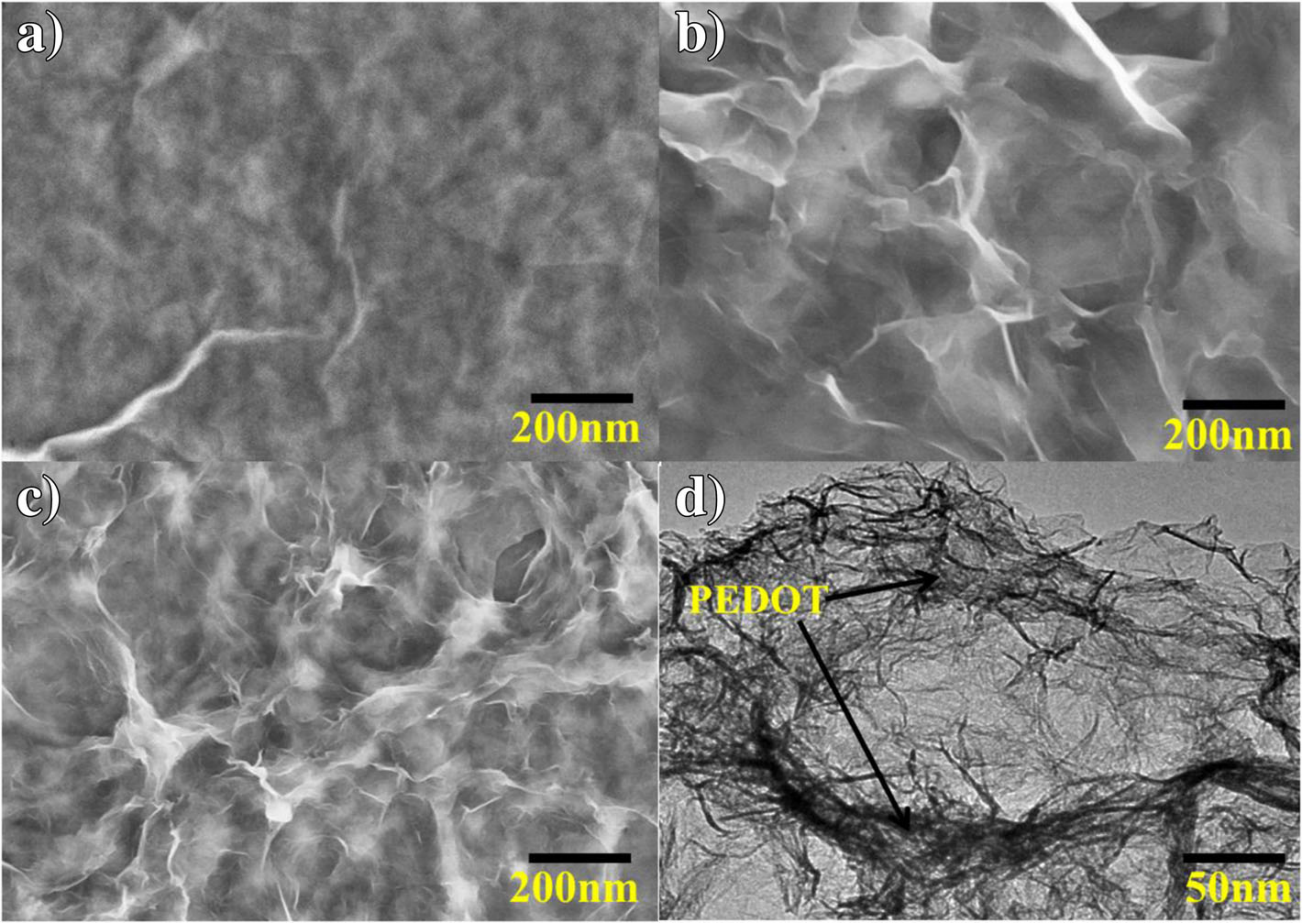
SEM images of **a** GO, **b** hydrothermal-treated rGO, **c** hydrothermal-polymerized rGO/PEDOT, and **d** TEM image of rGO/PEDOT

Figure 6 shows the SEM images of hydrothermal-treated rGO and rGO/PEDOT. After the hydrothermal treatment, the obtained rGO exhibits the typical wrinkle morphology (as shown in Fig. 6b), which shows no distinct difference to the GO sheets (as shown in Fig. 6a). As for rGO/PEDOT composite, the morphology with rGO sheets covered by PEDOT particle is presented (as shown in Fig. 6c), which is also confirmed by the TEM analysis (as shown in Fig. 6d). These results reveal that the PEDOT particles are successfully anchored on rGO sheets through an oxidant-free hydrothermal polymerization method. The EDS analysis also confirms the anchoring of PEDOT layer on rGO sheets with the evidence that the S and O element distribution is presented in the composite layer.

Due to the reduction of GO into rGO and anchoring highly conductive PEDOT on rGO sheets, the obtained rGO/PEDOT composite would be beneficial for enhanced conductivity. Table 1 shows the conductivity of GO, rGO, and rGO/PEDOT after the hydrothermal treatment. An obvious enhancement of conductivity is achieved after the hydrothermal stirring, indicating the partly reduction of GO into rGO. Moreover, after the hydrothermal reaction with the addition of EDOT monomer, the obtained composite shows the drastic enhancement of conductive capability. Compared with hydrothermal-treated rGO, almost 4 orders of magnitude of conductivity increase are observed in rGO/PEDOT, due to the thorough reduction of GO and covering of highly conductive PEDOT on rGO sheets. Table 1 also indicates that the mass ratio of GO shows distinct influence on the conductive performance of obtained composite and an optimized addition of 4 g GO results in highest conductivity, which results from efficient polymerization of EDOT and anchoring of PEDOT on rGO.

**Table 1:** Conductivity of GO, hydrothermal-treated GO, and hydrothermal-polymerized rGO/PEDOT

Samples	GO	rGO	rGO/PEDOT1	rGO/PEDOT2	rGO/PEDOT3	rGO/PEDOT4
Conductivity (S/cm)	×	3.5 ± 1.5 × 10^−4^	8.3 ± 0.1	20.7 ± 0.4	88.5 ± 0.5	61.7 ± 0.5

**Fig. 7:**
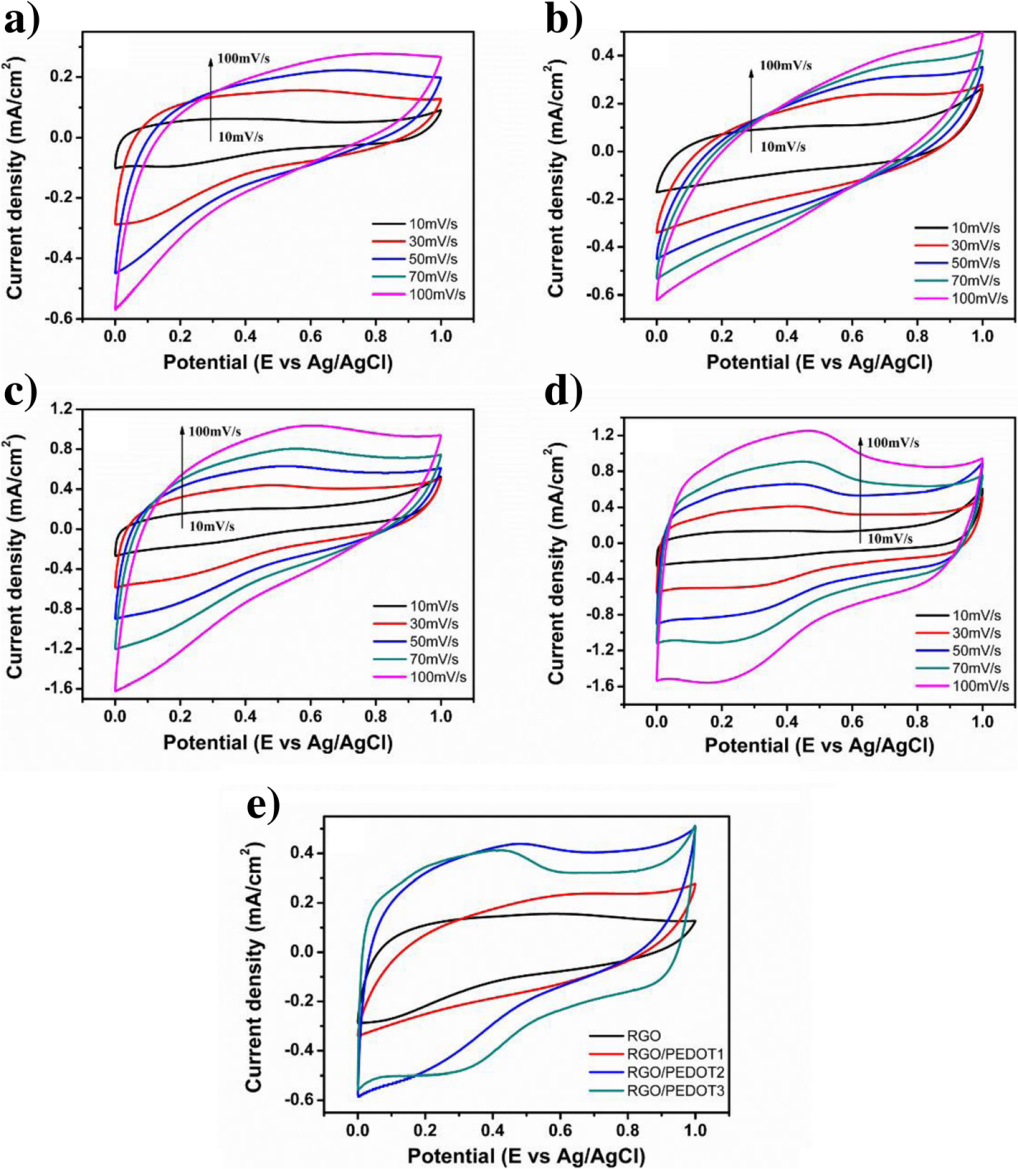
CV curves of **a** rGO, **b** rGO/PEDOT1, **c** rGO/PEDOT2, and **d** rGO/PEDOT3 at different scanning voltage rate, and **e** compared CV curve of rGO with different rGO/PEDOT at 30 mV/s scanning voltage rate

This highly conductive composite can be deposited on different substrate easily through spraying or spin-coating methods as electrode materials and the electrochemical performances are evaluated. Figure 7 shows the cyclic voltammetry (CV) curves of hydrothermal-treated rGO and hydrothermal-polymerized rGO/PEDOT electrodes (Fig. 7a–d). It can be seen that a distinct increase of CV area was achieved after the modification of PEDOT on rGO sheets. Both the pseudocapacitance of PEDOT and electric double layer capacitance (EDLC) of rGO attribute to the total capacitance of composite electrodes. With the increase of scanning rate, the relative increase of CV cure area is observed, indicating the excellent capacitance performance of composite electrodes. Figure 7e shows the CV curves of rGO/PEDOT composite electrodes prepared with different GO/EDOT mass ratios. It has been shown that with the increase of GO contents at a constant content of EDOT monomer, the obtained rGO/PEDOT electrodes present larger specific capacitance (SC) due to more GO are reduced into rGO and the EDOT monomer polymerized into PEDOT more efficiently. This result is also consistent with conductive performance investigation and indicates that proper GO content in hydrothermal reaction needs to be optimized to obtain composite with both good conductive and electrochemical performance.

**Fig. 8:**
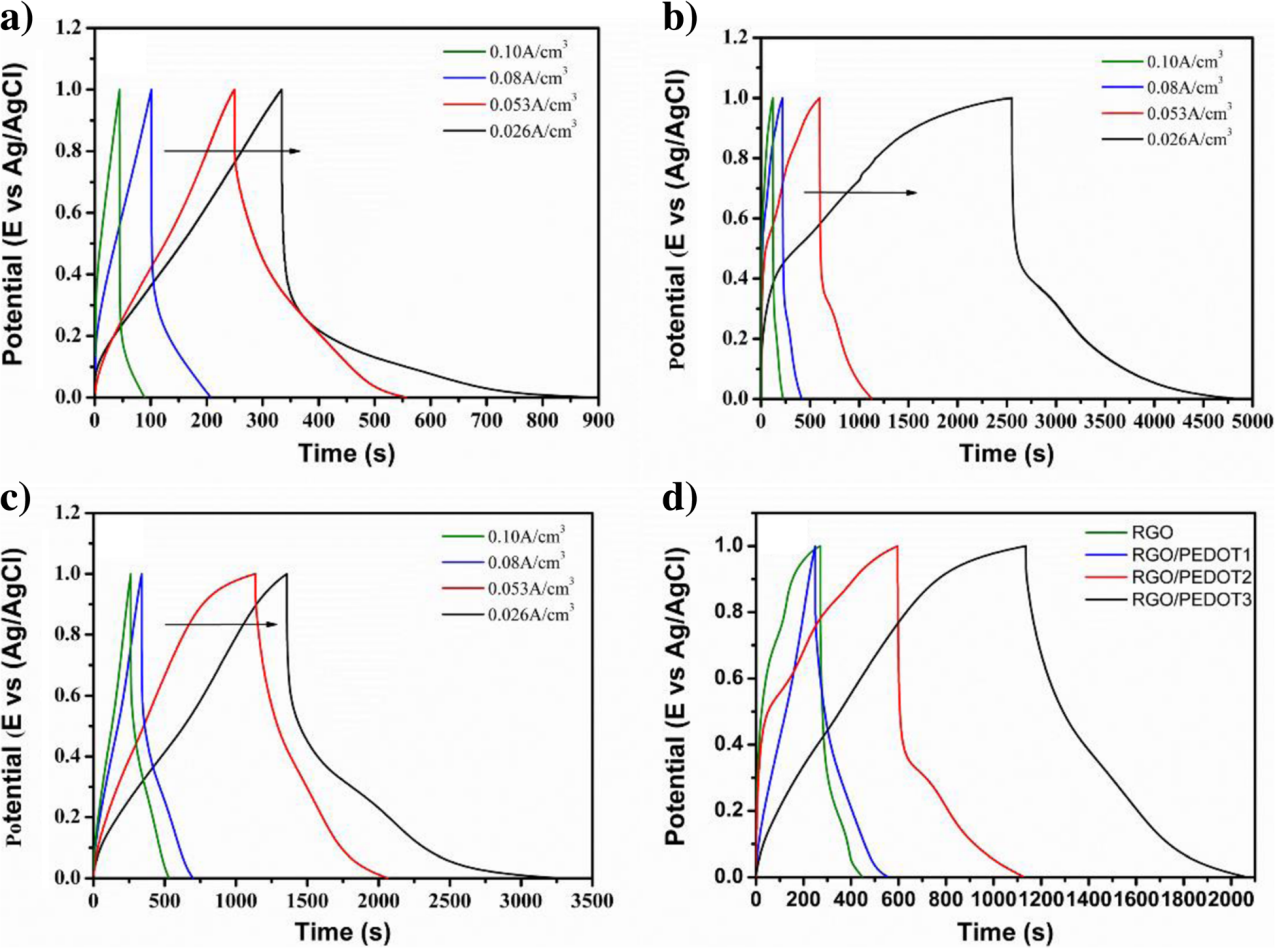
GCD curves of **a** rGO, **b** rGO/PEDOT2, and **c** rGO/PEDOT3 with the increasing of scanning current density. **d** GCD curves of rGO and different rGO/PEDOT electrodes at a 0.053 A/cm^3^ scanning current density

Constant current charging and discharging (GCD) curves of different rGO/PEDOT electrodes are also tested, which are shown in Fig. 8a–c. With the increase of scanning current density, the relative increase of GCD cure area is observed, indicating the excellent charge/discharge performance of composite electrodes. The GCD performance of composite electrodes also shows a GO content-dependent relationship, which is shown in Fig. 8d. With the increase of GO contents during the hydrothermal polymerization, the increasing GCD curve area of obtained composite electrodes is observed, indicating the enhanced energy storage performance can be achieved at an optimized GO mass ratio during the hydrothermal reaction, which shows the same tendency with CV performance.

Table 2 shows the calculated specific capacitance (SC) of rGO and different rGO/PEDOT electrodes. From the table, we can see that the proper ratio of GO mass shows a distinct influence on SC performance of obtained composite electrodes. The SC of composite electrode increases from 120.5 F/g to 202.7 F/g with the GO mass increasing from 1.5 g to 4 g, which is reasonable for more thorough reduction of GO and production of polymerized PEDOT. When the GO mass ratio was increased to 4.5 g, a reduced SC of composite electrode about 183.3F/g was presented. This result is good which consists of the conductive investigation presented in Table 1. We conclude that the excessive addition of GO resulted in distinct aggregation and the reduction of specific capacitance of composite electrode occurred accordingly. After the anchoring of PEDOT particle on the rGO sheets at the proper GO concentration, the introduction of PEDOT pseudocapacitance would enhance the specific capacitance obviously than pure rGO electrode. Our composite electrodes show higher specific capacitance (SC) than reported pure rGO electrodes with varied architecture [64, 65], our previously reported rGO/PEDOT composite electrode prepared through an oxidant polymerization method [66], composite rGO/PEDOT:PSS electrodes [67, 68], composite rGO/Polypyrrole (PPY) [69], and our PEDOT/rGO electrode obtained from physical mixing (Table 3). These electrodes also show comparable SC performance with a high SC rGO/polyaniline (PANi) electrode [70], but show higher conductivity which is a benefit to construct high power density devices. Furthermore, as porous electrode materials, our composite electrode exhibits a 66.5F cm^−3^ volumetric capacitance (VC), which was comparable for the high VC electrode constructed from graphene aerogel/conducting polymers [71].

**Table 2:** Calculated specific capacitance of different electrodes

Samples	rGO	rGO/PEDOT1	rGO/PEDOT2	rGO/PEDOT3	rGO/PEDOT4
Specific capacitance (F/g)	12.3	120.5	168.9	202.7	183.3

**Table 3:** Comparison of our work with reported literatures

Ref	Method	Materials	Performance		
			Specific capacitance	Capacitance retention	Volumetric capacitance
[52]	Electrochemistry coating	rGO	151 F/g at 1 A/g	98% after 500 cycles	N/A
[53]	Dip coating	rGO	180 F/	N/A	N/A
[54]	In situ polymerization	PEDOT-PSS/RGO	193.7 F/g at 500 mA/g	90.6% after 1000 cycles	N/A
[55]	Physical mixing	PEDOT-PSS/RGO	448 mF/cm^2^ at 10 mV/s	N/A	49.9 F cm^−3^
[56]	Physical mixing	MnO^2^/rGO/PEDOT:PSS	139.7 F/g at 1A/g	66.2% after 2000 cycles	N/A
[57]	Physical mixing	rGO/PEDOT:PSS	12.3 F/g at 5 mV/s	200% stretching	N/A
[58]	Chemical polymerization	PANi-g-rGO	250 F/g	N/A	N/A
Our work	Hydrothermal polymerization method	rGO/PEDOT	202.7 F/g at 1 A/g	90% after 9000 cycles	66.5 F cm^−3^
Our work	Physical mixing	rGO and purchased PEDOT	166.3 F/g at 1 A/g	77% after 3000 cycles	24.2 F cm^−3^
[71]	Chemical modification	Graphene aerogel/conducting polymer	N/A	N/A	28~180 F cm^−3^

**Fig. 9:**
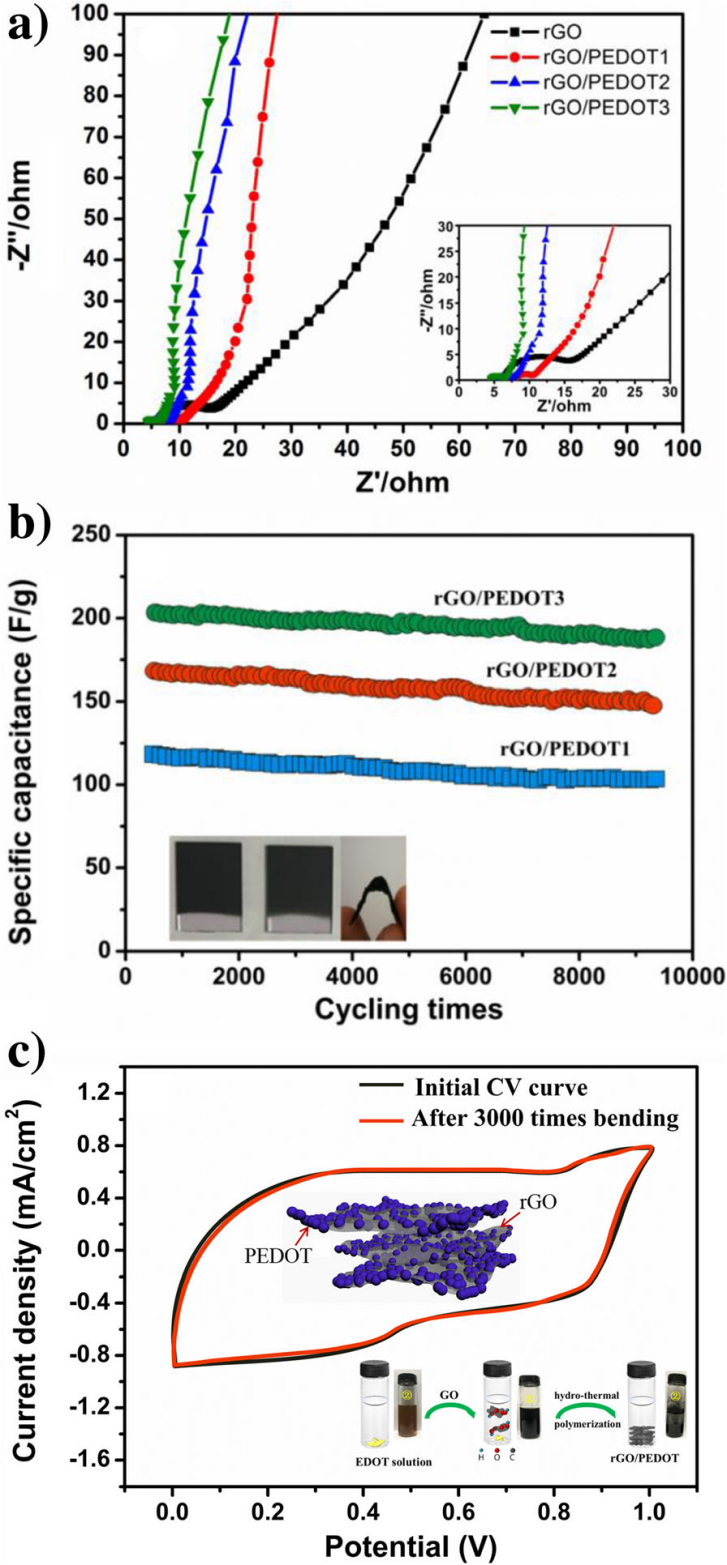
**a** EIS performance of hydrothermal-treated GO and hydrothermal-polymerized rGO/PEDOT. **b** Cycling performance of rGO/PEDOT electrodes at a 1.0 A/g scan current density, the insets are optical picture of rGO/PEDOT deposited on ITO and cotton fabric. **c** Capacitance reliability of rGO/PEDOT3 flexible electrode

Figure 9 a shows the Nyquist plots of rGO and different rGO/PEDOT composite electrodes. It can be seen that the composite electrodes show smaller inner resistant (Rs) than pure rGO. The smallest Rs electrode comes from the optimized hydrothermal reaction under optimized GO/EDOT mass ratio, which can trigger the polymerization of EDOT efficiently and reduction of GO thoroughly, and the obtained electrodes exhibit higher conductive capability. This Rs result is also well consistent with the conductivity result of different electrodes mentioned above. The rate performance of composite rGO/PEDOT electrodes and rGO prepared through hydrothermal reaction is also evaluated, and this performance is vital for actual application for energy storage applications. As shown in Fig. 9b, the rGO/PEDOT electrodes exhibit good cycling ability, and the specific capacitance maintained above 90% after more than 9000 times cycle at a 1.0 A/g scan current density. As we know that the pure conductive polymer will exhibit poor electrochemical stability after long time cycling, our rGO/PEDOT composite electrodes show good stability after long time cycles. We attribute this to the excellent mechanical strength of rGO, which affords stable support for PEDOT polymer during charging/discharging process. Furthermore, this composite electrode also shows excellent flexible ability, which is characterized and shown in Fig. 9c. It can be seen that more than 95% of the initial capacitance of electrode maintains after three thousands of free bending, which shows promising performance to construct flexible energy storage devices.

## Conclusions

The rGO/PEDOT nanocomposite has been successfully prepared by a hydrothermal polymerization method. The ultrathin conducting PEDOT layer was constructed on rGO sheets through an oxidant-free polymerization method in aqueous GO solution. In this hydrothermal reaction, the functional groups on the GO sheets trigger the polymerization of EDOT, and the GO are reduced to rGO accordingly. The results of conductive and electrochemical performance indicated that the PEDOT-anchored rGO exhibited high conductivity and showed enhanced energy storage capability than pure hydrothermal-prepared rGO. Furthermore, the excellent mechanical ability of rGO affords robust support for conducting PEDOT simultaneously during the energy storage process. This highly conductive nanostructure of conducting polymer PEDOT on rGO shows promising application for high-performance electrochemical electrodes and flexible devices.

## Data Availability

All datasets are presented in the main paper or in the additional supporting files.

## Abbreviations

CPs: Conducting polymers
CV: Cyclic voltammetry
EDLC: Electric double layer capacitance
EDOT: 3,4-ethylenedioxythiophene
EDOT: Polymerization of 3,4-ethylenedioxythiophene
EIS: Electrochemical impedance spectroscopy
GCD: Galvanostatic charge–discharge
GO: Graphene oxide
PANi: Polyaniline
PEDOT: Poly (3,4-ethylenedioxythiophene)
PPY: Polypyrrole
PSS: Poly (styrenesulfonate)
rGO: Reduced graphene oxide
Rs: Resistant
SC: Specific capacitance

## References

[1] Gunes S, Neugebauer H, Sariciftci NS (2007). Conjugated polymer-based organic solar cells. Chem Rev.

[2] Liu RY, Wang J, Sun T, Wang MJ, Wu CS, Zou HY, Song T, Zhang XH, Lee ST, Wang ZL, Sun BQ (2017). Silicon nanowire/polymer hybrid solar cell-supercapacitor: a self-charging power unit with a total efficiency of 10.5%. Nano Lett.

[3] Chen JD, Cui CH, Li YQ, Zhou L, Ou QD, Li C, Li YF, Tang JX (2015). Single-junction polymer solar cells exceeding 10% power conversion efficiency. Adv Mater.

[4] Sharma S, Hussain S, Singh S, Islam SS (2014). MWCNT-conducting polymer composite based ammonia gas sensors: a new approach for complete recovery process. Sens Actuator B-Chem.

[5] Baker CO, Huang XW, Nelson W, Kaner RB (2017). Polyaniline nanofibers: broadening applications for conducting polymers. Chem Soc Rev.

[6] Huynh TP, Sharma PS, Sosnowska M, D'Souza F, Kutner W (2015). Functionalized polythiophenes: recognition materials for chemosensors and biosensors of superior sensitivity, selectivity, and detectability. Prog Polym Sci.

[7] Wang CL, Dong HL, Hu WP, Liu YQ, Zhu DB (2012). Semiconducting pi-conjugated systems in field-effect transistors: a material odyssey of organic electronics. Chem Rev.

[8] Chen T, Qiu JH, Zhu KJ, Li J, Wang JW, Li SQ, Wang XL (2014). Ultra high permittivity and significantly enhanced electric field induced strain in PEDOT:PSS–RGO/PU intelligent shape-changing electro-active polymers. RSC Adv.

[9] Shi Y, Peng L, Ding Y, Zhao Y, Yu G (2015). Nanostructured conductive polymers for advanced energy storage. Chem Soc Rev.

[10] Shi Y, Peng LL, Yu GH (2015). Nanostructured conducting polymer hydrogels for energy storage applications. Nanoscale.

[11] Li L, Wu Z, Yuan S, Zhang XB (2014). Advances and challenges for flexible energy storage and conversion devices and systems. Energy Environ Sci.

[12] Gerard M, Chaubey A, Malhotra BD (2002). Application of conducting polymers to biosensors. Biosens Bioelectron.

[13] Balint R, Cassidy NJ, Cartmell SH (2014). Conductive polymers: towards a smart biomaterial for tissue engineering. Acta Biomater.

[14] Yang G, Kampstra KL, Abidian MR (2014). High-performance conducting polymer nanofiber biosensors for detection of biomolecules. Adv Mater.

[15] Shi Y, Pan LJ, Liu BR, Wang YQ, Cui Y, Bao ZA, Yu GH (2014). Nanostructured conductive polypyrrole hydrogels as high-performance, flexible supercapacitor electrodes. J Mater Chem A.

[16] Wang K, Wu HP, Meng YN, Wei ZX (2014). Conducting polymer nanowire arrays for high performance supercapacitors. Small.

[17] Meng QF, Cai KF, Chen YX, Chen LD (2017). Research progress on conducting polymer based supercapacitor electrode materials. Nano Energy.

[18] Kurra N, Wang RQ, Alshareef HN (2015). All conducting polymer electrodes for asymmetric solid-state supercapacitors. J Mater Chem A.

[19] Beidaghi M, Gogotsi Y (2014). Capacitive energy storage in micro-scale devices: recent advances in design and fabrication of micro-supercapacitors. Energy Environ Sci.

[20] Jiang WC, Yu DS, Zhang Q, Goh KL, Wei L, Yong YL, Jiang RR, Wei J, Chen Y (2015). Ternary hybrids of amorphous nickel hydroxide-carbon nanotube-conducting polymer for supercapacitors with high energy density, excellent rate capability, and long cycle life. Adv Funct Mater.

[21] Yu ZN, Tetard L, Zhai L, Thomas J (2015). Supercapacitor electrode materials: nanostructures from 0 to 3 dimensions. Energy Environ Sci.

[22] Yang CY, Shen JL, Wang CY, Fei HJ, Bao H, Wang GC (2014). All-solid-state asymmetric supercapacitor based on reduced graphene oxide/carbon nanotube and carbon fiber paper/polypyrrole electrodes. J Mater Chem A.

[23] Li PX, Yang YB, Shi EZ, Shen QC, Shang YY, Wu ST, Wei JQ, Wang KL, Zhu HW, Yuan Q, Cao AY, Wu DH (2014). Core-double-shell, carbon nanotube/polypyrrole/MnO_2_ sponge as freestanding, compressible supercapacitor electrode. ACS Appl Mater Interfaces.

[24] Yang YJ, Yuan WT, Li SB, Yang XJ, Xu JH, Jiang YD (2015). Manganese dioxide nanoparticle enrichment in porous conducting polymer as high performance supercapacitor electrode materials. Electrochim Acta.

[25] Yu M, Ma YX, Liu JH, Li SM (2015). Polyaniline nanocone arrays synthesized on three-dimensional graphene network by electrodeposition for supercapacitor electrodes. Carbon.

[26] Yun TG, Hwang BI, Kim D, Hyun S, Han SM (2015). Polypyrrole-MnO_2_-coated textile-based flexible-stretchable supercapacitor with high electrochemical and mechanical reliability. ACS Appl Mater Interfaces.

[27] Cong HP, Ren XC, Wang P, Yu SH (2013). Flexible graphene-polyaniline composite paper for high-performance supercapacitor. Energy Environ Sci.

[28] Snook GA, Kao P, Best AS (2011). Conducting-polymer-based supercapacitor devices and electrodes. J Power Sources.

[29] Al-Saleh MH, Sundararaj U (2009). A review of vapor grown carbon nanofiber/polymer conductive composites. Carbon.

[30] Yu GH, Xie X, Pan LJ, Bao ZN, Cui Y (2013). Hybrid nanostructured materials for high-performance electrochemical capacitors. Nano Energy.

[31] Ghenaatian HR, Mousavi MF, Rahmanifar MS (2012). High performance hybrid supercapacitor based on two nanostructured conducting polymers: self-doped polyaniline and polypyrrole nanofibers. Electrochim Acta.

[32] Hussain AM, Kumar A (2006). Enhanced electrochemical stability of all-polymer redox supercapacitors with modified polypyrrole electrodes. J Power Sources.

[33] Zhang JT, Zhao XS (2012). Conducting polymers directly coated on reduced graphene oxide sheets as high-performance Supercapacitor electrodes. J Phys Chem C.

[34] Laforgue A (2011). All-textile flexible supercapacitors using electrospun poly (3,4-ethylenedioxythiophene) nanofibers. J Power Sources.

[35] Wu Q, Xu YX, Yao ZY, Liu AR, Shi GQ (2010). Supercapacitors based on flexible graphene/polyaniline nanofiber composite films. ACS Nano.

[36] Lehtimaki S, Suominen M, Damlin P, Tuukkanen S, Kvarnstrom C, Lupo D (2015). Preparation of supercapacitors on flexible substrates with electrodeposited PEDOT/graphene composites. ACS Appl Mater Interfaces.

[37] Anothumakkool B, Soni R, Bhange SN, Kurungot S (2015). Novel scalable synthesis of highly conducting and robust PEDOT paper for a high performance flexible solid supercapacitor. Energy Environ Sci.

[38] Frackowiak E, Khomenko V, Jurewicz K, Lota K, Beguin F (2006). Supercapacitors based on conducting polymers/nanotubes composites. J Power Sources.

[39] Wang J, Xu YL, Chen X, Du XF (2007). Electrochemical supercapacitor electrode material based on poly (3,4-ethylenedioxythiophene)/polypyrrole composite. J Power Sources.

[40] Tong LY, Liu J, Boyer SM, Sonnenberg LA, Fox MT, Ji DS, Feng J, Bernier WE, Jones WE (2017). Vapor-phase polymerized poly (3,4-ethylenedioxythiophene) (PEDOT)/TiO_2_ composite fibers as electrode materials for supercapacitors. Electrochim Acta.

[41] D'Arcy JM, El-Kady MF, Khine PP, Zhang LH, Lee SH, Davis NR, Liu DS, Yeung MT, Kim SY, Turner CL, Lech AT, Hammond PT, Kaner RB (2014). Vapor-phase polymerization of nanofibrillar poly (3,4-ethylenedioxythiophene) for supercapacitors. ACS Nano.

[42] Chen Y, Xu JH, Yang YJ, Zhao YT, Yang WY, Mao XL, He X, Li SB (2016). The preparation and electrochemical properties of PEDOT:PSS/MnO2/PEDOT ternary film and its application in flexible micro-supercapacitor. Electrochim Acta.

[43] Lawal AT, Wallace GG (2014). Vapour phase polymerisation of conducting and non-conducting polymers: a review. Talanta.

[44] Brooke R, Cottis P, Talemi P, Fabretto M, Murphy P, Evans D (2017). Recent advances in the synthesis of conducting polymers from the vapour phase. Prog Mater Sci.

[45] Zhu Y, James DK, Tour JM (2012). New routes to graphene, graphene oxide and their related applications. Adv Mater.

[46] Kang SM, Park SJ, Kim D, Park SY, Ruoff RS, Lee H (2011). Simultaneous reduction and surface functionalization of graphene oxide by mussel-inspired chemistry. Adv Funct Mater.

[47] Harshal PM, Gupta K, Singh R, Sharma OP, Sugimura H, Khatri OP (2019). Alkylated graphene oxide and reduced graphene oxide: grafting density, dispersion stability to enhancement of lubrication properties. J Colloid Interface Sci.

[48] Bagri A, Mattevi C, Acik M, Chabal YJ, Chhowalla M, Shenoy VB (2010). Structural evolution during the reduction of chemically derived graphene oxide. Nat Chem.

[49] Khandelwal M, Seung HH, Chung JS (2019). Tailoring the structural properties of simultaneously reduced and functionalized graphene oxide via alkanolamine(s)/alkyl alkanolamine for energy storage applications. Chem Eng J.

[50] Mehdi SN, Fatemeh Z (2017). Electrochemical reduced graphene oxide-polyaniline as effective nanocomposite film for high-performance supercapacitor applications. Electrochim Acta.

[51] Lorena CV, ZaragozaContreras EA, Vega–Rios A (2017). Synthesis of graphene oxide/poly (3,4–ethylenedioxythiophene) composites by Fenton’s reagent. Polymer.

[52] Zarrin N, Tavanai H, Abdolmaleki A, Bazarganipour M, Alihosseini F (2018). An investigation on the fabrication of conductive polyethylene dioxythiophene (PEDOT) nanofibers through electrospinning. Synth Met.

[53] Wang XW, Zhang ZA, Yan XL, Qu YH, Lai YQ, Li J (2015). Interface polymerization synthesis of conductive polymer/graphite oxide@sulfur composites for high-rate lithium-sulfur batteries. Electrochim Acta.

[54] Fan MM, Zhu CL, Liu L, Wu QL, Hao QL, Yang JZ, Sun DP (2016). Modified PEDOT by benign preparing N-doped reduced graphene oxide as potential bio-electrode coating material. Green Chem.

[55] Xu KL, Chen GM, Qiu D (2013). Convenient construction of poly (3,4-ethylenedioxythiophene)-graphene pie-like structure with enhanced thermoelectric performance. J Mater Chem A.

[56] Diez-Pascual AM, Sanchez JAL, Capilla RP, Diaz PG (2018). Recent developments in graphene/polymer nanocomposites for application in polymer solar cells. Polymers.

[57] Ramesha GK, Sampath S (2009). Electrochemical reduction of oriented graphene oxide films: an in situ Raman spectroelectrochemical study. J Phys Chem C.

[58] Kim KH, Kim JY, Kim KB (2012). Facile coating of poly (3,4-ethylenedioxythiophene) on manganese dioxide by galvanic displacement reaction and its electrochemical properties for electrochemical capacitors. Bull Kor Chem Soc.

[59] Du FP, Cao NN, Zhang YF, Fu P, Wu YG, Lin ZD, Shi R, Amini A, Cheng C (2018). PEDOT:PSS/graphene quantum dots films with enhanced thermoelectric properties via strong interfacial interaction and phase separation. Sci Rep.

[60] Lindfors T, Noeva ZA, Latonen RM (2014). Electrochemical synthesis of poly (3,4-ethylenedioxythiphene) in aqueous dispersion of high porosity reduced graphene oxide. RSC Adv.

[61] Majeed Maitham H., Shayesteh Payam, Persson Axel R., Wallenberg L. Reine, Schnadt Joachim, Wendt Ola F. (2018). A PdII Carbene Complex with Anthracene Side-Arms for π-Stacking on Reduced Graphene Oxide (rGO): Activity towards Undirected C-H Oxygenation of Arenes. European Journal of Inorganic Chemistry.

[62] Spanninga SA, Martin DC, Chen Z (2010). X-ray photoelectron spectroscopy study of counterion incorporation in poly (3,4-ethylenedioxythiophene) (PEDOT) 2: polyanion effect, toluenesulfonate, and small anions. J Phys Chem C.

[63] Yang DX, Velamakanni A, Bozoklu G, Park S, Stoller M, Piner RD, Stankovich S, Jung I, Field DA, Ventrice CA, Ruoff RS (2009). Chemical analysis of graphene oxide films after heat and chemical treatments by X-ray photoelectron and micro-Raman spectroscopy. Carbon.

[64] Xu YH, Li J, Huang WX (2017). Porous graphene oxide prepared on nickel foam by electrophoretic deposition and thermal reduction as high-performance supercapacitor electrodes. Materials.

[65] Ramabadran U, Ryan G, Zhou X, Farhat S, Manciu F, Tong Y, Ayler R, Garner G (2017). Reduced graphene oxide on nickel foam for supercapacitor electrodes. Materials (Basel, Switzerland).

[66] Yang WY, Zhao YT, He X, Chen Y, Xu JH, Li SB, Yang YJ, Jiang YD (2015). Flexible conducting polymer/reduced graphene oxide films: synthesis, characterization, and electrochemical performance. Nanoscale Res Lett.

[67] Liu YQ, Weng B, Razal JM, Xu Q, Zhao C, Hou YY, Seyedin S, Jalili R, Wallace GG, Chen J (2015). High-performance flexible all-solid-state supercapacitor from large free-standing graphene-PEDOT/PSS films. Sci Rep.

[68] Yan D, Liu Y, Li YH, Zhuo RF, Wu ZG, Ren PY, Li SK, Wang J, Yan PX, Geng ZR (2014). Synthesis and electrochemical properties of MnO_2_/rGO/PEDOT:PSS ternary composite electrode material for supercapacitors. Mater Lett.

[69] Zhao C, Shu KW, Wang CY, Gambhir S, Wallace GG (2015). Reduced graphene oxide and polypyrrole/reduced graphene oxide composite coated stretchable fabric electrodes for supercapacitor application. Electrochim Acta.

[70] Kumar NA, Choi HJ, Shin YR, Chang DW, Dai LM, Baek JB (2012). Polyaniline-grafted reduced graphene oxide for efficient electrochemical supercapacitors. ACS Nano.

[71] Hong JY, Wie JJ, Xu Y, Park HS (2015). Chemical modification of graphene aerogels for electrochemical capacitor applications. Phys Chem Chem Phys.

